# Neuromuscular junction dysfunctions due to immune checkpoint inhibitors therapy: An analysis of FAERS data in the past 15 years

**DOI:** 10.3389/fimmu.2022.778635

**Published:** 2022-08-22

**Authors:** Ping Zhang, Donghui Lao, Haoyan Chen, Bin Zhao, Qiong Du, Qing Zhai, Xuan Ye, Bo Yu

**Affiliations:** ^1^ Department of Pharmacy, Tongren Hospital, Shanghai Jiao Tong University, School of Medicine, Shanghai, China; ^2^ Department of Pharmacy, Zhongshan Hospital, Fudan University, Shanghai, China; ^3^ Department of Pharmacy Pharmacology, Peking Union Medical College Hospital, Chinese Academy of Medical Science and Peking Union Medical College, Beijing, China; ^4^ School of Medicine, Fudan University, Shanghai, China; ^5^ Department of Pharmacy, Fudan University Shanghai Cancer Center, Shanghai, China

**Keywords:** immune checkpoint inhibitor, neuromuscular junction dysfunction, adverse event, data mining, FAERS

## Abstract

**Introduction:**

The adverse effects of neuromuscular junction dysfunctions caused by immune checkpoint inhibitor (ICI) drugs have not been thoroughly assessed in the clinics.

**Objective:**

To assess the neuromuscular junction dysfunctions in cancer patients with adverse events caused by ICI therapy by searching the Food and Drug Administration Adverse Event Reporting System (FAERS) database.

**Methods:**

The FAERS data from January 2004 to December 2020 were collected to analyze the association between neuromuscular connection dysfunction and ICI use. Disproportionate analysis and Bayesian analysis were used to quantify the association between the neuromuscular junction dysfunctions and ICIs. The onset time and outcome of neuromuscular junction dysfunctions in different ICI regimens were also compared.

**Results:**

Out of 88,617 adverse event reports, 557 neuromuscular junction dysfunction reports (0.63%) were analyzed. Marketed ICI drugs, including ipilimumab, nivolumab, pembrolizumab, atezolizumab, durvalumab, cemiplimab, avelumab, as well as their combinations, showed positive associations with four detection methods. Most of the adverse event reports were associated with the use of nivolumab (53.32%) and pembrolizumab (31.96%). However, nivolumab-related neuromuscular junction dysfunctions were similar with pembrolizumab (33.33% vs 33.14%, p > 0.05). The onset time of neuromuscular junction dysfunctions showed no significant difference among different ICIs (p > 0.05).

**Conclusions:**

Analysis of FAERS data identified that over 30% (32.85%) of reports of neuromuscular junction dysfunctions resulted in death. Ongoing monitoring, risk evaluations, and further comparative studies of ICIs should be considered.

## Introduction

The development of immune checkpoint inhibitors (ICIs) is a revolutionary milestone in immune-oncology. ICIs revive the anti-tumor immune responses by interrupting the co-inhibitory signaling pathways and promoting immune-mediated tumor cell clearance (1). Immune-related adverse events (irAEs) from ICIs differ from toxicities caused by cytotoxic or targeted therapy agents. The onset of toxicities may be delayed and may not follow the periodic pattern observed with conventional cytotoxic drugs. The mechanism of toxicity remains to be determined and may even vary between patients receiving the same drug. The over-reactive immune response may be due to ICI exposure to low levels of autoreactive T cells, macrophage-mediated toxicity, or the elimination of tolerance caused by the production of antibodies from activated B cells (2). These irAEs have a wide range of affected organs and severity. Skin, endocrine, neurological, gastrointestinal, respiratory, and musculoskeletal toxicity may occur alone or in combination. Most events are self-limiting or can be resolved with immunosuppressive agents (such as corticosteroids) (3). Persistent irAEs that cannot be resolved with corticosteroids require tumor necrosis factor-alpha receptors antagonists, such as infliximab, an inhibitor of purine synthesis in T and B cells. Only a few irAEs don’t respond to these immune modulators (4). Numerous randomized controlled trials provide a rough overview of immune-related adverse events, including skin, gastrointestinal, lung, liver, and endocrine toxicity (5). Although most immune-related adverse events can be well controlled by supportive treatment and glucocorticoids, fatal immune-related adverse events have received increasing attention regarding the safety and patient tolerance of ICI drugs (6, 7).

ICI-related neurological adverse events are relatively infrequent. However, pooled analyses on these events show severe morbidity and mortality (8, 9). These irAEs can affect any level of the peripheral nervous system, including peripheral nerves, neuromuscular junctions, and muscles, individually or in combination (10). The adverse effects of neuromuscular junction dysfunctions caused by different ICIs have not been thoroughly assessed in the clinics. Recently, some reports updated the information on neuromuscular junction dysfunctions (11–14). Since detailed pathological mechanisms, more effective treatments, as well as rechallenge strategies remain obscure, early detection and data on the outcomes are of great importance (11, 13). Pharmacovigilance (PV) systems have been established for side effect (ADR) monitoring by countries or organizations such as the World Health Organization (WHO), the European Medicines Agency (EMA), and the US Food and Drug Administration (FDA). The FDA’s Adverse Event Reporting System (FAERS) is the world’s largest repository of reported hazardous drug events (15). Reporting all adverse events to the FDA depends on healthcare professionals, consumers, and manufacturers (16). The purpose of the system is to support the FDA safety monitoring of pharmaceuticals and therapeutic biologics on the market. In the present study, we collected, screened, and statistically analyzed FAERS data and performed signal mining analysis focusing on possible correlations for neuromuscular junction dysfunctions generated by ICI.

## Materials and methods

### Data source

We conducted a retrospective pharmacovigilance study using data from the FAERS database (https://fis.fda.gov/extensions/FPD-QDE-FAERS/FPD-QDE-FAERS.html) covering the period from January 2004 to December 2020. We conducted a retrospective pharmacovigilance survey. FAERS is a public, voluntary reporting database that provides information on adverse events and drug error reports submitted by healthcare professionals, consumers, or pharmaceutical companies. The data is not only from the United States but also from other countries. The FAERS data file contains demographic and administrative details, drug information, the Medical Dictionary for Regulatory Activities’ (MedDRA) preferred terminology for adverse event (REAC) coding, patient outcomes, reporting sources, initiation of treatment that includes date and end date reports, and indications for use. A deduplication procedure was performed according to the FDA’s recommendations of selecting the latest FDA_DT when the CASEIDs were the same and selecting the higher PRIMARYID when the CASEID and FDA_DT were the same. A total of 88,617 reports related to ICIs were mined from the FAERS database, and 557 reports (0.63%) were associated with neuromuscular junction dysfunction.

### Adverse events and drug identification

Adverse events were identified using the MedDRA terms “Recurrence of neuromuscular blockade (10068408)”, “Ocular myasthenia (10049168)”, “Neuromuscular blockade (10029315)”, “Neuromuscular block prolonged (10029314)”, “Myasthenic syndrome (10028424)”, “Myasthenia gravis neonatal (10028419)”, “Myasthenia gravis crisis (10062758)”, “Myasthenia gravis (10028417)”, in the REAC file. The drugs in the FAERS database can be registered using different conventions; therefore, the MICROMEDEX^®^ (Index Nominum) was used as a dictionary for ICIs. Reports involving the seven immunotherapies for cancer treatment (including ipilimumab, nivolumab, pembrolizumab, cemiplimab, atezolizumab, avelumab, and durvalumab) were identified using text string searches for each drug by generic names, brand names, and abbreviations. Reports listing one or more of therapies mentioned above as the primary suspect (PS) or secondary suspect (SS) agent were included. We excluded drugs reported with the eligible drug listed as interacting or concomitant, as well as those without cancer-related indications.

### Data mining

Based on disproportionality analysis and Bayesian analysis, 4 algorithms were applied: reporting odds ratio (ROR), the proportional reporting ratio (PRR), the Bayesian confidence propagation neural network, and the multi-item gamma Poisson shrinker. In combination, these algorithms were used to identify the association between drugs and adverse events. **Table 1** shows the equations and criteria for the four algorithms (17–26). The use of concomitant agents is considered in the current analysis to calculate an adjusted disproportionality (adjusted ROR) which is used in the current study if not otherwise stated. We evaluated the onset time and mortality of each immune checkpoint inhibitor regimen. Onset time was defined as the interval between EVENT_DT (the date of occurrence of adverse events) and START_DT (start date of use of immune checkpoint inhibitor). It did not contain a date entry containing an input error (EVENT_DT before START_DT) and an incorrect date. Reports of fatal events due to drug toxicity were compiled, and mortality was calculated as the number of fatal events divided by the total number of neuromuscular junction dysfunction associated with each ICI regimen.

**Table 1:** Summary of major algorithms used for signal detection.

Algorithms	Equation	Criteria
ROR	*ROR*=(*a*/*b*)/(*c*/*d*)	95% CI>1, N≥2
PRR	*PRR*=(*a*/(*a* + *c*))/(*b*/(*b* + *d*)) *χ*2=*Σ*((*O*−*E*)2/*E*); (*O*=*a*, *E*=(*a*+*b*)(*a*+*c*)/(*a*+*b*+*c*+*d*)	PRR≥2, χ2≥4, N≥3
BCPNN	*IC*=*log*2*a*(*a*+*b*+*c*+*d*)/((*a*+*c*)(*a*+*b*))	IC025>0
MGPS	*EBGM*=*a*(*a*+*b*+*c*+*d*)/((*a*+*c*)(*a*+*b*))	EBGM05>2, N>0

a, number of reports containing both the suspect drug and the suspect adverse drug reaction; b, number of reports containing the suspect adverse drug reaction with other medications (except the drug of interest); c, number of reports containing the suspect drug with other adverse drug reactions (except the event of interest); d, number of reports containing other medications and other adverse drug reactions. ROR, reporting odds ratio; CI, confidence interval; N, the number of co-occurrences; PRR, proportional reporting ratio; BCPNN, Bayesian confidence propagation neural network; IC, information component; IC025, the lower limit of the 95% two-sided CI of the IC; MGPS, multi-item gamma Poisson shrinker; EBGM, empirical Bayesian geometric mean; EBGM05, the lower 95% one-sided CI of EBGM.

### Handling missing data and removal of duplicates

A deduplication procedure was performed as described by firstly linking CASE number with relevant ISRs, secondly through the 4-key fields used in missing data handling (event_date, age, gender, reporter_country) and thirdly revising drug and event reported and the single imputation approach was performed to manage missing data (27).

### Statistical analysis

Descriptive analyses were used to summarize the clinical features of the patients with neuromuscular junction dysfunctions collected from the FAERS database. Time to onset of neuromuscular junction dysfunctions between different ICI regimens was compared using nonparametric tests when the data were not normally distributed (Mann-Whitney test for dichotomous variables, the Kruskal-Wallis test when there were more than two subgroups of respondents). Pearson’s chi-square test or Fisher’s exact test was used to comparing outcome events and fatality rates between different ICI regimens. Statistical significance was determined with 95% CI and p<0.05. Data mining and statistical analyses were performed with SAS, version 9.4 (SAS Institute Inc., Cary, NC, USA).

## Results

### General characteristics

Out of 88,617 adverse reports, 557 neuromuscular junction dysfunction reports (0.63%) were documented in the FAERS database with ICIs (**Table 2**). Most of the 557 reports were related to nivolumab (53.32%%) and pembrolizumab (31.96%). **Table 3** presents the clinical characteristics of patients.

**Table 2:** Two-by-two contingency table for report collection and database analysis.

	Reports with a drug of interest	Reports without a drug of interest	Total reports
Reports with an adverse event of interest	557	90,076	90,633
Reports without an adverse event of interest	88,060	271,157,873	271,245,933
Total reports	88,617	271,247,949	271,336,566

Drug of interest: ICIs (ipilimumab, nivolumab, pembrolizumab, atezolizumab, durvalumab, cemiplimab, avelumab, as well as combinations. Adverse event of interest: Neuromuscular junction dysfunction, including recurrence of neuromuscular blockade, ocular myasthenia, neuromuscular blockade, neuromuscular block prolonged, myasthenic syndrome, myasthenia gravis neonatal, myasthenia gravis crisis, myasthenia gravis.

**Table 3:** Clinical characteristics of patients.

	Ipilimumab	Nivolumab	Pembrolizumab	Cemiplimab	Atezolizumab	Avelumab	Durvalumab	Nivo+ipi^a^	Pembro+ipi^b^	Atezo+Ipi^c^	Total
Patient age, years											
Mean	67.09	70.63	70.96	88.00	71.19	70.25	68.38	68.37	66.67		70.20
Not reported	3	38	34	6	5		3	20		2	111
Patient gender											
Male	8	63	52	1	13	2	10	23	2		174
Female	6	119	116		18	2	12	60	1		334
Not reported		19	7	6			2	13		2	49
Reporting region											
Africa		1									1
Asian	3	73	52		9	1	7	38			183
Europe	3	41	29	1	12	2	5	17			110
Oceania		4	15				2				21
North America	8	82	77	6	10	1	10	39	3	2	238
South America			2					2			4
Reporters											
Consumer	5	20	79	1			1	6			112
Other health- professional	4	48	16	1		1	3	23	3		99
Pharmacist		23	11		1		3	9			47
Physician	4	89	58	4	28	3	12	46			244
Not reported	1	21	11	1	2		5	12		2	55
Indications											
Breast cancer			2		1		1	1			5
Genitourinary cancers	1	40	11		2	1	1	50			106
Gastric Cancer		13	2								15
Pancreatic cacer				1	1						2
Liver cancer		3	2		1		1				7
Colorectal cancer		5	1					2			8
Hepatobiliary Cancer		3	2		1		1				7
Gynecologic cancer		1	3		1	1					6
Head and neck cancers		4	1	1			1	1			8
Lung Cancer	1	64	54		14	1	16	14	1	2	167
Thoracic cancer		2	7					2			11
Melanoma	11	40	49		1			22	2		125
Neuroendocrine carcinoma		2	1			1	1				5
Lymphoma		2		1							3
Haematological malignancy								1			1
Unknown	1	16	17		10		1	3			48
Other		1	10	3							14
Not reported		2	14	1			2				19
Total	14	201	175	7	31	4	24	96	3	2	557

a Nivolumab+Ipilimumab.

b Pembrolizumab+Ipilimumab.

c Atezolizumab+Ipilimumab.

The number of neuromuscular junction dysfunction events was the highest for nivolumab (n=201), followed by pembrolizumab (n=175). The mean age of these patients was slightly over 70 years. Most of the cases of neuromuscular junction dysfunctions involved with ICIs were reported from North America, Asia, and Europe (42.73%, 32.85%, and 19.75%, respectively). Most of the cases were submitted by physicians for nivolumab (44.28%) and by consumers for pembrolizumab (45.14%), and the cases appeared more often in females than in males (59.96% vs. 31.24%, respectively).

### Signal detection

All ICI were associated with neuromuscular junction dysfunction, and signal detection was positive among all four methods (**Table 4**). Cemiplimab had a higher score than other ICIs in single-drug therapies. Nivolumab+Ipilimumab had a higher score than other combinations of ICIs.

**Table 4:** Signal detection.

All regimens	N	ROR	PRR	IC	EBGM
		(95% two-sided CI)	(χ2)	(IC025)	(EBGM05)
Ipilimumab	14	4.41 (2.61,7.46)	4.41 (36.74)	2.14 (1.26)	4.39 (2.83)
Nivolumab	201	18.55 (16.1,21.38)	18.45 (3163.91)	4.14 (3.59)	17.64 (15.66)
Pembrolizumab	175	27.48 (23.61,31.97)	27.25 (4269.72)	4.71 (4.05)	26.18 (23.06)
Cemiplimab	7	41.96 (19.89,88.53)	41.4 (275.6)	5.37 (2.54)	41.33 (22.13)
Atezolizumab	31	12.52 (8.78,17.83)	12.47 (324.75)	3.63 (2.55)	12.39 (9.21)
Avelumab	4	15.07 (5.64,40.27)	15 (52.24)	3.91 (1.46)	14.99 (6.58)
Durvalumab	24	24.78 (16.83,36.5)	24.59 (585)	4.61 (3.13)	24.45 (17.68)
Nivo+Ipi^a^	96	23.83 (19.45,29.19)	23.65 (2036.73)	4.53 (3.7)	23.15 (19.53)
Pembro+Ipi^b^	3	23.46 (7.53,73.08)	23.28 (63.95)	4.54 (1.46)	23.27 (8.99)
Atezo+Ipi^c^	2	193.17 (46.21,807.38)	181.52 (359)	7.5 (1.8)	181.44 (54.83)

a Nivolumab+Ipilimumab.

b Pembrolizumab+Ipilimumab.

c Atezolizumab+Ipilimumab.

### Onset time of events

In general, the median time to event onset of ICI-associated neuromuscular junction dysfunctions was 27 days. Onset time was significantly shorter with pembrolizumab than atezolizumab (*p*=0.006) and durvalumab (*p*=0.019); similar results were found in nivolumab (*p*=0.005 compared to atezolizumab and *p*=0.019 compared to durvalumab). However, when combined with ipilimumab, nivolumab led to a significantly faster onset of events than atezolizumab (*p*= 0.037) (**Table 5**).

**Table 5:** Onset time (days).

	Ipilimumab^1^	Nivolumab^2^	Pembrolizumab^3^	Cemiplimab^4^	Atezolizumab^5^	Avelumab^6^	Durvalumab^7^	Nivo+Ipi^a,8^	Pembro+Ipi/^b,9^
n	8	119	108	5	24	2	10	56	1
Median	46.5	26	24	22.5	38	459	44	6.5	53
Mean	57.67	27.92	40.44	24.5	78.42	459	44.63	48.86	53
SE	56.15	35.34	83.07	5.94	102.62	432	15.88	88.24	0
First quartile	22.5	17	13.75	19.75	28.5	243	36	18	53
Third quartile	60	34	39.25	27.25	95.5	675	57.5	42	53

The onset time p values between groups were compared using nonparametric tests (Kruskal-Wallis test). 3 vs. 5: p=0.006; 3 vs. 7: p=0.019; 2 vs. 5: p=0.005; 2 vs. 7: p=0.019; 8 vs. 5: p= 0.037; p > 0.05 between other groups.

a Nivolumab+Ipilimumab.

b Pembrolizumab+Ipilimumab.

The numbers 1-9 represent the different drug groups. The onset time p values between groups were compared using nonparametric tests (Kruskal-Wallis test).

### Outcome events and incidence

To determine the prognosis of neuromuscular junction dysfunctions after ICI treatments, we assessed neuromuscular junction dysfunctions following usage of ICIs (**Table 6**). It was observed that neuromuscular junction dysfunctions generally led to hospitalizations for nivolumab and pembrolizumab (68.16% and 69.71%, respectively). Among the 557 reports, 32.85% (183 out of 557) led to death. When comparing most frequently reported agents, nivolumab exhibits similar risks with or without ipilimumab. The outcomes of pembrolizumab shows no difference in terms of death, life-threatening disability, and other serious events compared to nivolumab.

**Table 6:** Outcome events.

	Ipilimumab	Nivolumab	Pembrolizumab	Cemiplimab	Atezolizumab	Avelumab	Durvalumab	Nivolumab+Ipilmimuab^a^	Pembrolizumab+Ipilimumab^b^	Atezolizumab+Ipilimumab^c^
Total reports	14	201	175	7	31	4	24	96	3	2
Outcome events n (%)										
Death	2(14.29)	67(33.33)	58(33.14)	3(42.86)	12(38.71)	1(25.00)	9(37.50)	30(31.25)	(0)	1(50.00)
Life-Threatening	4(4.17)	80(39.80)	39(22.29)	2(28.57)	2(6.45)	(0)	3(12.50)	23(23.96)	(0)	(0)
Disability	1(1.04)	13(6.47)	20(11.43)	(0)	(0)	2(2.08)	1(4.17)	2(2.08)	(0)	(0)
Other Serious (Important Medical Event)	10(10.42)	194(96.52)	158(90.29)	5(71.43)	13(41.94)	2(2.08)	7(29.17)	92(95.83)	2(66.67)	1(50.00)
Hospitalization - Initial or Prolonged	11(11.46)	137(68.16)	122(69.71)	5(71.43)	20(64.52)	3(3.13)	14(58.33)	64(66.67)	1(33.33)	(0)
Required Intervention to Prevent Permanent Impairment/Damage	1(1.04)	(0)	2(1.14)	(0)	(0)	(0)	(0)	(0)	(0)	(0)
Impairment/Damage										

^a^Nivolumab+Ipilimumab.

^b^Pembrolizumab+Ipilimumab.

^c^Atezolizumab+Ipilimumab.

## Discussion

Our study concludes that though quite rare (0.63% of all reports), ICIs lead to severe adverse reactions (**Table 6**) on neuromuscular junction dysfunctions. In the current analysis, nivolumab and pembrolizumab account for most of the reports (201 + 175 = 376 vs. 557). These adverse events may be more easily discovered when ICI-drugs are prescribed in the larger patient population in the near future. However, monitoring programs must be present to help detect these rare but potentially severe side effects and provide an essential basis for future prevention.

Drugs that target the PD-L1/PD-1 pathway may exhibit a more favorable toxicity profile than CTLA4 blockade and conventional chemotherapy (28). However, our results confirm that ICIs targeting the PD-1 pathway show a higher risk of neuromuscular junction dysfunctions than ICIs targeting the CTLA4 pathway.

CTLA4 is an immune checkpoint molecule expressed on immune cells that contains both CD4+ and CD8+ T cell subsets and B cells. CTLA4 antibody (ipilimumab) can restore T cells priming and activation by tumor antigens and promote antitumor T cell response. The PD-L1/PD-1 interaction is another checkpoint for anti-cancer immunity that functions downstream of T cell priming and activation. PD-1 is expressed on the surface of activated T cells, but its specific ligand PD-L1 is present in multiple tissue types, including many other different cancer cells. When PD-L1 binds to PD-1, it inhibits T cell proliferation and activity and reduces cytokine production, helping malignant cells evade the host’s immune response. Therapeutic antibodies designed to target PD-L1/PD-1 interactions include antibodies that inhibit PD-1 (nivolumab and pembrolizumab) or PD-L1 (atezolizumab).

The clinical presentation of neuromuscular junction dysfunctions is often atypical, with considerable overlap between myasthenia gravis and myopathy, as well as cardiac/respiratory complications. However, mortality was high in these patients, despite adequate treatment strategies including corticosteroid. Several pathogenic mechanisms, including neuronal damage by T cells and autoantibodies and/or cytokine-mediated inflammation processes, have been hypothesized. However, the pathogenesis of these ICI-related complications is not completely understood (14). Clinical and experimental immune-mediated neuropathy studies have shown that autoimmune responses to peripheral nervous tissues are not limited to dense myelin along the main nerve trunk. They can also affect the nerve body, the axonal structures of the node of Ranvier, and the neuromuscular junction. Types of ICI-related neuropathy include demyelinating neuropathy, axonal sensorimotor polyneuropathy, pure sensory axonal neuropathy, and mononeuropathy multiplex based on electrophysiological studies (29).

Choosing a fast and effective signal detection method provides a valuable signal for drug risk management, which can severely impact human health and minimize harm to humans. However, risks are difficult to detect only through experimental studies with limited sample size. Therefore, this study provides a reference to confirm the occurrence of neuromuscular junction dysfunctions associated with ICIs through post-marketing surveillance studies.

Healthcare professionals treating cancer patients are advised to be aware of Guillain-Barre Syndrome (GBS) symptoms. These patients need immediate attention and should be given a low threshold for hospitalization to facilitate work-up and monitor severe or life-threatening symptoms (30). Currently, limited sources are comparing ICI-associated neuromuscular junction dysfunctions between PD-1 and PD-L1 targeting pathways. Signal detection suggested that pembrolizumab, either without or with ipilimumab, showed a higher score than other ICIs or ICI combinations. Our results also showed that ICIs targeting PD-1 (nivolumab and pembrolizumab) exhibited more neuromuscular junction dysfunctions than ICIs targeting the PD-L1 pathway (avelumab, atezolizumab, durvalumab, and semiprimal). However, there could be confounding factors such as a limited sample size since agents targeting the PD-L1 pathway entered the market later.

While the data mining techniques used in this study have many advantages as a tool for detecting signs of adverse drug reaction, it should also be noted that this technology does not solve all problems in detecting and analyzing signs of adverse drug reaction. The signals from FAERS were used only for qualitative research.

This study has certain limitations. First of all, data mining technology cannot completely reflect all clinical information from patients. Detailed information from clinical follow-ups and other studies are also needed to validate the signals detected. Secondly, data mining technology cannot address ADR reporting system issues such as inaccuracy, false reporting, incomplete reporting, underreporting, and arbitrariness. Thirdly, qualitative research makes it difficult to quantify the side effect signals of neuromuscular junction dysfunctions from a few ADRs. Finally, the restrictions of limited sample size cannot be overlooked.

## Conclusion

Immune checkpoint inhibitors need to be prescribed more cautiously. Analysis of FAERS data showed that over 30% of reports of neuromuscular junction dysfunctions resulted in death, emphasizing the importance of constant monitoring, risk assessment, and more comparative studies.

## Data availability statement

The original contributions presented in the study are included in the Article/supplementary material. Further inquiries can be directed to the corresponding authors.

## Author contributions

All contributors are listed as co-authors. BY and DL: conceptualization. QD and XY: methodology. BZ: data retrieving. DL: formal analysis and investigation. HC: writing original draft preparation. PZ: writing. QZ: review and editing. BY: supervision. All authors contributed to the article and approved the submitted version.

## Funding

This study was supported by the Chinese Pharmaceutical Association-Servier Youth Hospital Pharmaceutical Innovation Research Funding Project and Hospital Development Center of Shanghai Municipality (SHDC2020CR3085B).

## Conflict of interest

The authors declare that the research was conducted in the absence of any commercial or financial relationships that could be construed as a potential conflict of interest.

## Publisher’s note

All claims expressed in this article are solely those of the authors and do not necessarily represent those of their affiliated organizations, or those of the publisher, the editors and the reviewers. Any product that may be evaluated in this article, or claim that may be made by its manufacturer, is not guaranteed or endorsed by the publisher.
